# Application of one heartbeat acquisition with motion correction algorithm in CCTA of patients with atrial fibrillation: evaluation of coronary artery stenoses using artificial intelligence assisted diagnostic system

**DOI:** 10.3389/fradi.2025.1691838

**Published:** 2025-11-25

**Authors:** Shumeng Zhu, Xing Li, Qian Tian, Xiaoqian Jia, Tingting Qu, Jianying Li, Xueyan Zhang, Yannan Cheng, Le Cao, Lihong Chen, Jianxin Guo

**Affiliations:** 1 Department of Radiology, The First Affiliated Hospital of Xi’an Jiaotong University, Xi’an, Shaanxi, China; 2 CT Research Center, GE Healthcare China, Beijing, China

**Keywords:** atrial fibrillation, coronary artery CT angiography, artificial intelligence, diagnostic performance, motion correction algorithm

## Abstract

**Introduction:**

Motion artifacts induced by atrial fibrillation (AF) present a substantial challenge in coronary computed tomography angiography (CCTA). Wide detectors, rapid scanning, and motion correction algorithms can effectively improve image quality in CCTA. This study aims to evaluate the impact of one-beat acquisition with a motion correction algorithm (Snapshot Freeze 1, SSF1) on the image quality of prospective CCTA in patients with AF, and its diagnostic performance using an artificial intelligence assisted diagnostic system (AI-ADS).

**Materials and methods:**

A total of 91 consecutive patients with AF, who underwent one-beat CCTA were analyzed. Images were reconstructed with SSF1. The subjective and objective image quality of the coronary arteries were evaluated. Using the invasive coronary catheter angiography (ICA) as the reference standard, the diagnostic performance of AI-ADS and AI-ADS + radiologist for stenoses above moderate and severe degrees were calculated.

**Results:**

Effective radiation dose was 2.43 ± 0.88 mSv. The average CT values of all major coronary arteries and branches were greater than 400 HU. All vessels were diagnosable (scores ≥ 3) with good or above ratings at 96.15% (350/364) and 96.70% (352/364). The diagnostic accuracy, sensitivity, specificity and AUC of AI-ADS vs. AI-ADS + radiologist for above moderate stenoses were: (84.62% vs. 91.21%), (89.61% vs. 98.70%), (57.14% vs. 50.00%) and (0.73 vs. 0.74) on patient level; (84.07% vs. 87.64%), (74.07% vs. 85.19%), (89.96% vs. 89.08%) and (0.82 vs. 0.87) on vessel level; (90.84% vs. 93.11%), (63.59% vs. 78.34%), (95.99% vs. 95.91%) and (0.80 vs. 0.87) on segment level. For severe stenoses, these values were: (62.64% vs. 82.42%), (58.62% vs. 91.38%), (69.70% vs. 66.67%) and (0.64 vs. 0.79) on patient level; (82.97% vs. 89.29%), (46.43% vs. 75.00%), (93.93% vs. 93.57%) and (0.70 vs. 0.84) on vessel level; (92.23% vs. 95.16%), (36.92% vs. 66.92%), (98.06% vs. 98.14%) and (0.68 vs. 0.83) on segment level.

**Conclusion:**

One-beat CCTA with SSF1 provides high-quality coronary images for patients with AF. AI-ADS automatically distinguishes coronary images with different stenoses, but the sensitivity of AI-ADS is low, especially for severe stenoses. AI-ADS + radiologist further improves the diagnostic performance.

## 1 Introduction

Atrial fibrillation (AF) is a common arrhythmia in clinic (1). For paroxysmal AF and partial persistent AF that are ineffective to drug therapy, the guidelines both explicitly recommend catheter ablation as the first-line treatment (2). In addition, coronary heart disease (CAD) and AF have many same risk factors, and the two diseases often exist in combination (3); Furthermore, studies have found that the treatment and management of patients with AF combined with obstructive CAD are different from those of patients without obstructive CAD and the prognosis is poor (4). Preoperative coronary CT angiography (CCTA) enables the imaging of coronary vessels to identify obstructive CAD and the acquisition of three-dimensional anatomical structures of the left atrium and pulmonary veins to guide catheter ablation procedures. Therefore, it is of utmost importance for patients with AF to undergo CCTA before catheter ablation.

However, motion and step/slice misalignment artifacts caused by heart rate variability and high heart rate may significantly influence image quality and diagnostic performance in AF patients (5, 6), CCTA in AF patients is a more challenging application. The 256-slice, 16 cm wide-detector CT can complete CCTA in one-beat without being limited by heart rhythm or even respiration, and research have shown that patients with AF can obtain high-quality coronary artery images in the 256-slice CT combined with motion correction algorithm (Snapshot freeze 1, SSF1, GE Healthcare, America) (7, 8).

In recent years, deep convolutional neural network has significantly improved the CCTA image automatic segmentation and centerline extraction (9, 10), and speeded up the diagnosis of CAD through the artificial intelligence system of deep learning (11). Research has confirmed (12, 13) that the artificial intelligence assisted diagnostic system (AI-ADS) can significantly improve the work efficiency and maintain a high diagnostic accuracy for coronary artery stenoses, but there are relatively few studies focusing on the application of AI-ADS in the diagnosis of CAD in patients with AF.

Therefore, in this study, we intended to obtained CCTA images using one-beat acquisition combined with SSF1 and introduced AI-ADS to evaluate the application value of one-beat acquisition combined with SSF1 in CCTA of AF patients.

## 2 Materials and methods

### 2.1 General information

Patients diagnosed with AF who underwent CCTA before catheter ablation from July 2021 to December 2023 were included retrospectively. Patient exclusion criteria: (1) patients with coronary stent or coronary artery bypass grafting; (2) The indwelling needle was pre-embedded in the left forearm due to arteriovenous fistula or poor right vascular condition; (3) Patients with severe contrast agent allergy or renal insufficiency; (4) ICA was not performed within 2 weeks after CCTA examination due to various reasons. Finally, 91 patients were included. All patients were informed of the examination precautions before CCTA and signed an informed consent for the use of iodinated contrast medium.

### 2.2 Image acquisition method

#### 2.2.1 CCTA image acquisition method

All patients were scanned on a 256-slice, 16 cm wide-detector CT machine (Revolution CT, GE Healthcare, America). The prospective electrocardiogram-triggered axial scans were acquired while patients were breathing hold. The scanning range was from the level of tracheal bifurcation to the bottom of the heart. According to the size of the heart, the width of the detector of 140 mm or 160 mm was selected to minimize patient radiation dose. Images were collected within one heartbeat. Tube voltage was 100 kVp, with an automatic tube current modulation (range of 100–720 mA) to achieve a noise index of 21. The rotation speed was 0.28 s/r. Intelligent cardiac phase selection technique was used to automatically select the optimal cardiac phases for acquisition and reconstruction. If the initial scanner-provided reconstruction phase failed to meet the image evaluation requirements, the optimal phase would be manually re-selected. A total of 45–50 mL Iopromide (Bayer, Germany) was injected as the contrast medium with 370 mgI/ml concentration and at a flow rate of 4.5 mL/s, followed by an additional 30 mL of saline at the same flow rate. The bolus tracking technique was used to automatically start CCTA with the threshold set at 220 HU with the shortest delay (of about 3 s) after triggering. The region of interest (ROI) for monitoring the contrast agent was placed in the descending aorta with the plane of tracheal bifurcation. After data acquisition, the CCTA images were reconstructed with the reconstruction thickness and interval of 0.625 mm, adaptive statistical iterative reconstruction (ASIR-V) algorithm at 70%, and SSF1 to reduce coronary artery motion artifact.

#### 2.2.2 ICA image acquisition method

The digital subtraction angiography was carried out using Philips FD10 angiograph (Philips Corporation, Netherlands), and Omnipaque (GE Healthcare, America) was injected as the contrast medium with 350 mgI/mL concentration. The coronary artery catheter was inserted into the coronary artery through the femoral artery or the radial artery, and different angles were projected to identify the stenotic segment and degree of the coronary artery.

### 2.3 Image quality evaluation

#### 2.3.1 Subjective evaluation

A 5-point Likert scale was used to assess the image quality of the right coronary artery (RCA), left main coronary artery (LM), left anterior descending artery (LAD) and left circumflex artery (LCX). The detailed scales were: 5 points, excellent image quality for very confident diagnosis; Very clear outline of blood vessels; Good uniformity of vascular density; No motion artifact; 4 points, good image quality for full diagnosis; Good outline of blood vessel; Good uniformity of vascular density. Minimum motion artifacts; 3 points, Fair image quality not affecting diagnosis; Identifiable outline of blood vessels; Acceptable uniformity of vascular density; Mild motion artifacts; 2 points, Poor image quality affecting the diagnosis; Unclear vessel contours; Poor uniformity of vascular density; Obvious motion artifacts; 1 point, Poor image quality, impossible to make the diagnosis; Unclear outline of the blood vessels; Poor uniformity of vascular density; Heavy motion artifacts. All images were subjectively evaluated by two qualified radiologists (with 5 years and 10 years of experience in CT imaging), and the scores were subjected to consistency test. Images with a score of 3 points or more were considered to meet the diagnostic requirements.

#### 2.3.2 Objective evaluation

The CT value and standard deviation (SD) in vessel was measured by a radiologist (with 5 years of experience in CT imaging). The ROI was placed in the center of RCA, LM, LAD and LCX. The size of ROI was more than half of the blood vessel, avoiding calcification and pathological changes. The CT value and SD of intrapericardial fat was measured at the aortic root level, with the SD value used as the background noise, with an ROI area of 5 mm^2^, to avoid blood vessels, myocardium, and lesions. All ROIs were placed three times in different locations, and the average value was taken as the finally result. The sign-to-noise ratio (SNR) and contrast-to-noise ratio (CNR) were calculated for vessels:

$$SNR=\frac{CTvalue_{target\;vesssle}}{SD_{target\;vessle}}$$

$$CNR=\frac{CTvalue_{target\;vessle}-CTvalue_{\;fat}}{SD_{\;fat}}$$

### 2.4 Image processing and diagnosis

CCTA images were processed by AI-ADS (CoronaryDoc, ShuKun Techonolgy, Beijing) for diagnosing stenoses. Based on the CCTA axial images, the AI-ADS can automatically generate images such as volume rendering, curved surface reconstruction, vessel probe, and vessel straightening reconstruction to perform vascular extraction, stenoses detection and to automatically generate structured diagnostic reports. At the same time, a radiologist (10 years of experience in CT imaging) combined the original CCTA images, the AI-ADS processed images and the structured report to confirm or reject the results of AI-ADS and to give a final diagnosis of coronary artery.

AI-ADS and the radiologist both referred to the standards of SCCT (14) and adopted the 18-segment coronary artery segmentation method, and only evaluated the vessels with diameters larger than 1.5 mm. The stenoses were divided into mild stenoses (<50%), moderate stenoses (50%–70%) and severe stenoses (>70%) according to the degree of stenoses.

### 2.5 Deep learning model

The model is based on a CNN, which consists of neurons with learnable and adjustable weights and biases (15). A typical CNN consists of an input layer and an output layer, as well as one or more convolutional layers, pooling layers, and one or more fully connected layers. We first input a set of well-labeled CCTA images as a training set, where all vessels and lesions have already been correctly labeled manually, and then transfer the training set to a CNN, where the data are analyzed and learned. The CNN will automatically learn the basic rules and regulations for identifying vessels and lesions in traditional CCTA images. A well-trained CNN is able to obtain segmented images and stenoses degree predictions from the original images based on what is learned from the training set. In one of previous study, we trained a CNN to identify coronary branch vessels and coronary lumen from more than 10,000 CCTA cases as the coronary model.

### 2.6 Statistical analysis

The SPSS 23.0 statistical software was used for analysis. The data were expressed as mean ± SD. The consistency of subjective evaluation between observers was tested by kappa test, the Kappa consistency test (16): 0–0.2 represents poor consistency, 0.21–0.4 represents general consistency, 0.41–0.6 represents medium consistency, 0.61–0.8 represents good consistency, and 0.81–1.0 represents great consistency. Using ICA results as reference standard, the accuracy, sensitivity, specificity, positive predictive values and negative predictive values of AI-ADS and AI-ADS + radiologist in the diagnosis of coronary artery stenoses above moderate and severe were calculated. The diagnostic performances of AI-ADS and AI-ADS + radiologist for CAD were evaluated by receive operating characteristic curves and were quantitatively expressed with areas under the curve (AUCs). The diagnosis of stenoses were made at the patient level, the vascular level and the vascular segment level.

## 3 Results

### 3.1 General clinical data

A total of 91 patients were included, with the age of 67.04 ± 10.59 y, BMI of 24.31 ± 3.40 kg/m^2^, average heart rate displayed on ECG of 77.71 ± 21.03 beats/min, and heart rate variability of 25.43 ± 19.49 beats/min. The effective radiation dose for the patients was 2.43 ± 0.88 mSv. A total of 364 coronary vessels including 1365 segments were included. Based on ICA, 77 patients (84.62%) had above moderate stenoses and 58 patients (63.74%) had severe stenoses. Patient demographics and scanning parameters are shown in Table 1.

**Table 1:** Patient demographics and scanning parameters.

Basic information	Value
Age (y)	67.04 ± 10.59
Male [(*n*)%]	56 (62)
Height (m)	1.66 ± 7.38
Weight (kg)	67.04 ± 10.59
BMI (kg/m^2^)	24.31 ± 3.40
Comorbidities [*n* (%)]	
Diabetes mellitus	18 (20)
Hypertension	36 (40)
COPD	12 (13)
HR (beats/min)	
Average HR	77.71 ± 21.03
Maximum HR	118.69 ± 57.84
HR variability	25.43 ± 19.49
Radiation exposure	
CTDIvol (mGy)	11.09 ± 3.97
DLP (mGy**•**cm)	173.65 ± 62.96
ED (mSv)	2.43 ± 0.88

BMI, body mass index; COPD, chronic obstructive pulmonary disease; HR, heart rate; CTDIVol, CT dose index volume; DLP, dose-length product; ED, effective dose.

### 3.2 Image quality

**Figure 1:**
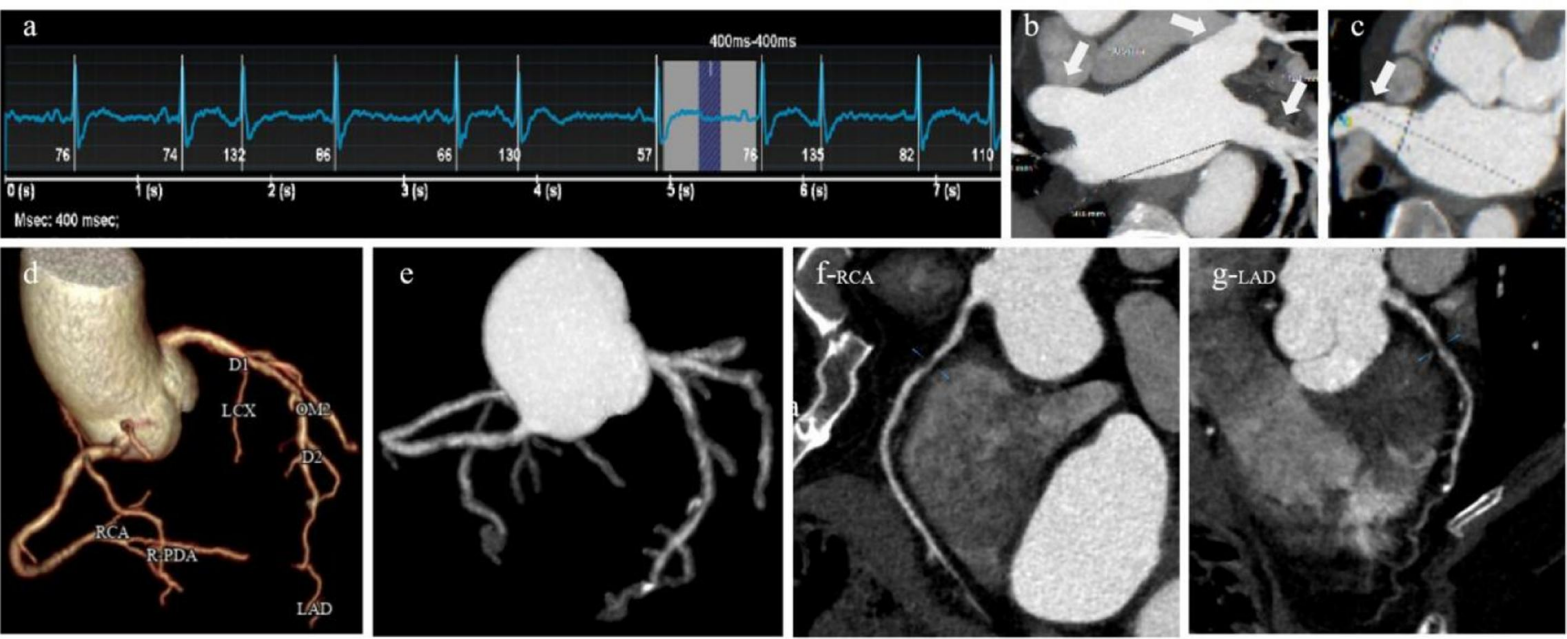
A 61-years-old atrial fibrillation patient. **(a)** ECG showed a heart rate of 82 beats/min and heart rate variability of 80 beats/min during the scan, **(b)** pulmonary vein, MIP, **(c)** left atrial appendage, MIP, **(d)** coronary artery tree, 3D-VR, **(e)** coronary artery tree, 3D-MIP, **(f)** right coronary artery, CPR, **(g)** left anterior descending coronary artery, CPR.

The average CT values of RCA, LM, LAD and LCX were all greater than 400HU; The CNR values of RCA, LM, LAD and LCX were 38.47 ± 7.49, 39.67 ± 7.59, 32.30 ± 13.64 and 37.24 ± 8.54, respectively. The two radiologists rated vessels as good (subjective score of 4 points) or above at 96.15% (350/364) and 96.70% (352/364), and the diagnosable rate reached 100% (subjective score ≥3 points). The interobserver consistency was very good, with the Kappa values greater than 0.8 (0.803–0.883), shown in Table 2 and Figure 1.

**Table 2:** Evaluation of subjective and objective image quality.

Coronary artery	RCA	LM	LAD	LCX
Objective measurement values				
CT value (HU)	433.88 ± 92.41	449.39 ± 79.89	420.63 ± 91.44	424.94 ± 96.22
SD_fat_ (HU)	21.00 ± 8.43	19.74 ± 7.10	18.73 ± 7.00	22.68 ± 8.58
SNR	24.32 ± 11.85	25.18 ± 8.42	25.74 ± 11.22	20.74 ± 8.01
CNR	38.47 ± 7.49	39.67 ± 7.59	32.30 ± 13.64	37.24 ± 8.54
Subjective evaluation results				
Reviewer 1 (5/4/3/2/1/)	64/21/6/0/0	87/4/0/0/0	62/24/5/0/0	71/17/3/0/0
Reviewer 2 (5/4/3/2/1/)	62/23/6/0/0	86/5/0/0/0	62/25/4/0/0	74/15/2/0/0
Consistency	0.878	0.883	0.881	0.803

RCA, right coronary artery; LM, left main coronary artery; LAD, left anterior descending branch; LCX, left circumflex branch; SD, standard deviation; SNR, signal-to-noise ratio; CNR, contrast-noise ratio.

### 3.3 Diagnostic accuracy for coronary artery stenoses

**Figure 2:**
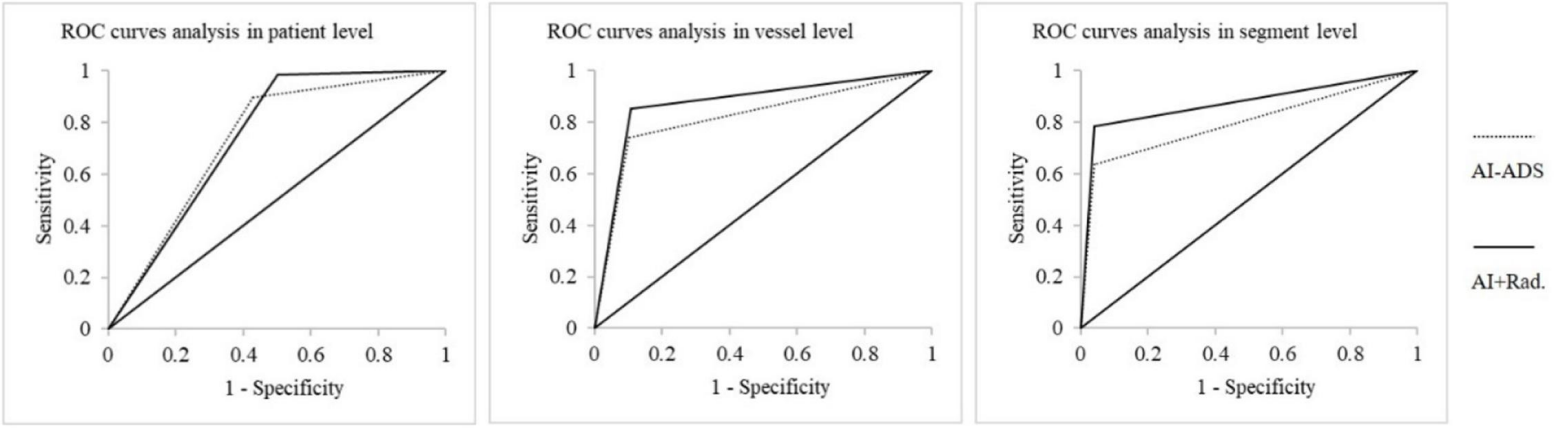
ROC curves for stenoses above moderate degree in patient, vessel and segment analyses.

**Figure 3:**
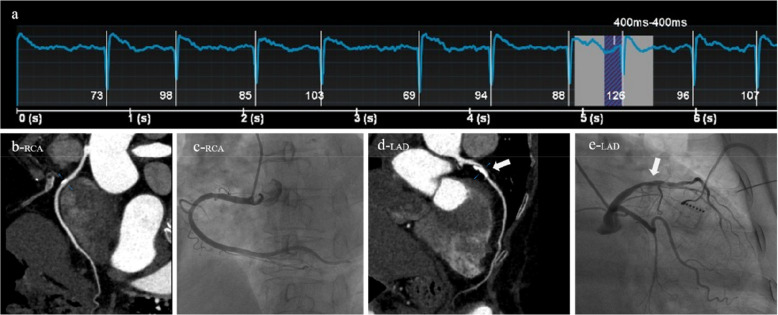
A 64-year-old female patient was clinically diagnosed with atrial fibrillation and arrhythmia. Both CT (AI-ADS, AI-ADS + radiologist) and ICA showed moderate stenoses of the sixth segment of the left anterior descending coronary artery, **(a)** ECG showed a heart rate of 96 beats/min and heart rate variability of 28 beats/min during the scan, **(b)** right coronary artery, CPR, **(c)** right coronary artery, ICA, **(d)** left anterior descending coronary artery, CPR, **(e)** left anterior descending coronary artery, ICA.

The diagnostic performances of AI-ADS vs. AI-ADS + radiologist for above moderate stenoses: diagnostic accuracy (84.62% vs. 91.21%), sensitivity (89.61% vs. 98.70%), and specificity (57.14% vs. 50.00%) on the patient level; (84.07% vs. 87.64%), (74.07% vs. 85.19%), and (89.96% vs. 89.08%) on the vessel level; (90.84% vs. 93.11%), (63.59% vs. 78.34%) and (95.99% vs. 95.91%) on the segment level. The diagnostic performances for AI-ADS + radiologist (AUCs were 0.74 for patient level, 0.87 for vessel level and 0.87 for segment level) were better than AI-ADS (0.73, 0.82 and 0.80). shown in Table 3, Figures 2, 3.

**Table 3:** Diagnostic results of stenoses above moderate degree.

Moderate stenosis	Total/positive	Method	TN	TP	FN	FP	Acc.%	Sen.%	Spec.%	PPV%	NPV%	AUC
Per-patient	91/77	AI-ADS	8	69	8	6	84.62	89.61	57.14	92.00	50.00	0.73
		AI + Rad.	7	76	1	7	91.21	98.70	50.00	91.57	87.50	0.74
Per-vessel	364/135	AI-ADS	206	100	35	23	84.07	74.07	89.96	81.30	85.48	0.82
		AI + Rad.	204	115	20	25	87.64	85.19	89.08	82.14	91.07	0.87
Per-segment	1,365/217	AI-ADS	1,102	138	79	46	90.84	63.59	95.99	75.00	93.31	0.80
		AI + Rad.	1,101	170	47	47	93.11	78.34	95.91	78.34	95.91	0.87

AI-ADS, artificial intelligence aided diagnosis system; Rad., radiologist; TN, true negative; TP, true positive; FN, false negative; FP, false positive; Acc., accuracy; Sen., sensitivity; Spec., specificity; PPV, positive predictive value; NPV, negative predictive value.

**Figure 4:**
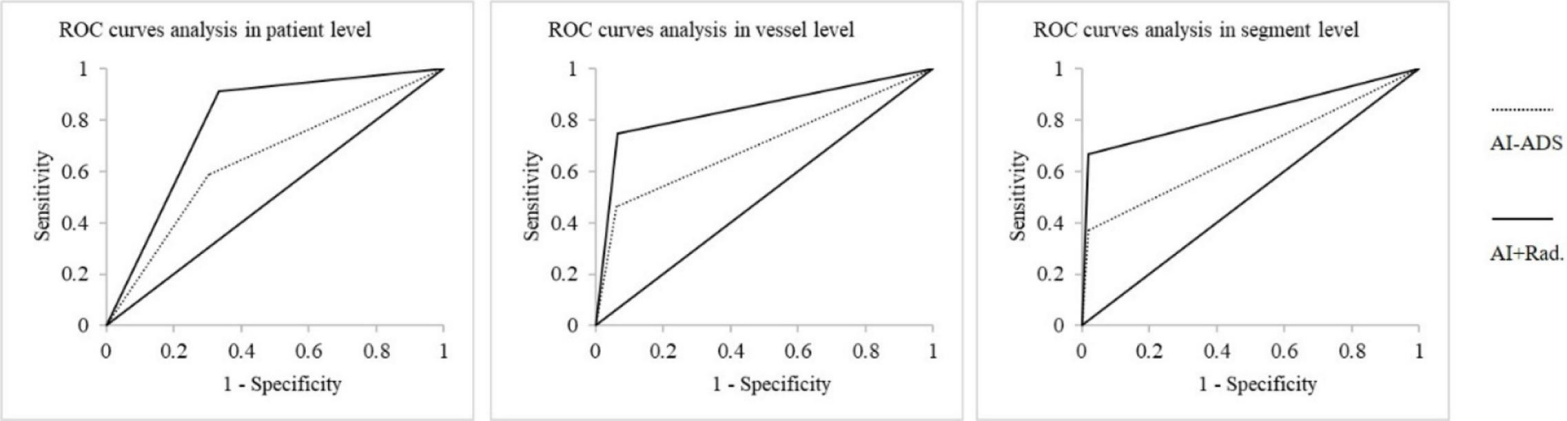
ROC curves for stenoses above severe degree in patient, vessel and segment analyses.

**Figure 5:**
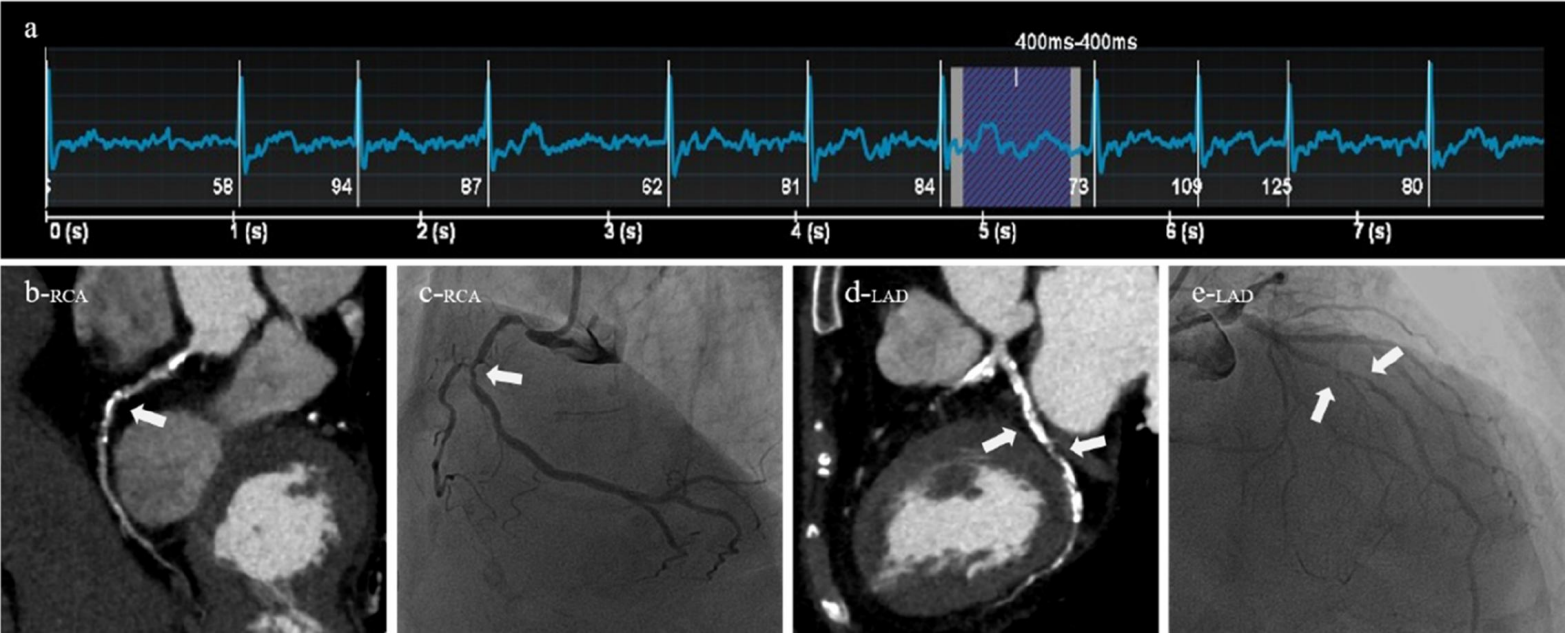
A 84-year-old male patient was clinically diagnosed with atrial fibrillation and arrhythmia. Both CT (AI-ADS, AI-ADS + radiologist) and ICA showed severe stenoses of the first segment of right coronary artery and the sixth and seventh segments of left anterior descending coronary artery, **(a)** ECG showed a heart rate of 84 beats/min and heart rate variability of 26 beats/min during the scan, **(b)** right coronary artery, CPR, **(c)** right coronary artery, ICA, **(d)** left anterior descending coronary artery, CPR, **(e)** left anterior descending coronary artery, ICA.

The diagnostic performances of AI-ADS vs. AI-ADS + radiologist for severe stenoses: diagnostic accuracy (62.64% vs. 82.42%), sensitivity (58.62% vs. 91.38%), and specificity (69.70% vs. 66.67%) on patient level; (82.97% vs. 89.29%), (46.43% vs. 75.00%), and (93.93% vs. 93.57%) on vessel level; (92.23% vs. 95.16%), (36.92% vs. 66.92%) and (98.06% vs. 98.14%) on segment level. The diagnostic performances for AI-ADS + radiologist (AUCs were 0.79 for patient level, 0.84 for vessel level and 0.83 for segment level) were better than AI-ADS (0.64, 0.70 and 0.68). shown in Table 4, Figures 4, 5.

**Table 4:** Diagnostic results of stenoses above severe degree.

Severe stenosis	Total/positive	Method	TN	TP	FN	FP	Acc.%	Sen.%	Spec.%	PPV%	NPV%	AUC
Per-patient	91/58	AI-ADS	23	34	24	10	62.64	58.62	69.70	77.27	48.94	0.64
		AI + Rad.	22	53	5	11	82.42	91.38	66.67	82.81	81.48	0.79
Per-vessel	364/84	AI-ADS	263	39	45	17	82.97	46.43	93.93	69.64	85.39	0.70
		AI + Rad.	262	63	21	18	89.29	75.00	93.57	77.78	92.58	0.84
Per-segment	1,365/130	AI-ADS	1,211	48	82	24	92.23	36.92	98.06	66.67	93.66	0.68
		AI + Rad.	1,212	87	43	23	95.16	66.92	98.14	79.09	96.57	0.83

AI-ADS, artificial intelligence aided diagnosis system; Rad., radiologist; TN, true negative; TP, true positive; FN, false negative; FP, false positive; Acc., accuracy; Sen., sensitivity; Spec., specificity; PPV, positive predictive value; NPV, negative predictive value.

## 4 Discusses

Patients with AF have rapid and irregular heart rates, which has always present challenges in the CCTA. In our study, wide-detector CT system was used to realize prospective cardiac CT acquisition in one heartbeat and CCTA images were corrected with SSF1 for cardiac motion. The results showed that, although the heart rate variability of patients in CCTA recorded by ECG was large, and the heart rate was high during the scan, high-quality coronary artery images were obtained at low radiation dose, and high diagnostic efficiencies for coronary stenoses were obtained with AI-ADS. In addition, before catheter ablation, CCTA examination simultaneously acquired anatomical structures such as the left atrium and pulmonary veins, further evaluated the left atrial appendage thrombosis, and provided richer clinical diagnostic information. Compared with ICA, which is invasive and has many complications, CCTA examination is undoubtedly a better choice for evaluating coronary artery diseases in patients with AF.

At present, CT examination is the largest medical radiation source (17). According to the literature, the dose of ionizing radiation received by patients with AF in CCTA examination fluctuated from 1.3–16 mSv (18, 19). In our study, the axial prospectively CT acquisition in one heartbeat was used, and the effective radiation dose was only 2.43 mSv, which was at the low dose level reported in the literature. The cardiac motion correction algorithm, SSF1 (8, 18, 20, 21), was used to effectively freeze the heart motion and significantly reduced motion artifacts. Andreini et al. (8) reported that the coronary artery images interpretability rate on a 256-slice CT combined with a motion correction algorithm in patients with atrial fibrillation was 98.1%. Zhang et al. (6) shown that in the preoperative TAVI evaluation of patients with atrial fibrillation using a motion correction algorithm, 99.7% of the vessel segments did not exhibit motion artifacts that would affect the diagnostic confidence. The subjective scoring results of CCTA in our study showed that the diagnosable rate reached 100% (subjective score ≥3 points), which is in an excellent with the previous studies. The motion artifact correction ability of SSF1 is reported to be affected by the CT value of vessels and previous studies have shown that the optimal vascular CT value for lesion detection by CCTA should be in the range of 350–450 HU (22). Achieving the optimal CT value can fully exert the advantages of SSF1, improve the quality of coronary artery and the confidence of radiologists. In our study, the average CT values of vessels were all in the range of 350–450 HU, providing adequate contrast enhancement in the vessels while minimizing the contrast dose requirement. The SNR and CNR of coronary artery images are basically consistent with those reported by Jia et al. (23) or even better than those reported by Zhang et al. (6) High SNR and CNR can improve radiologists' confidence in the diagnosis of coronary artery disease.

AI-ADS is a commonly used clinical software for CAD diagnosis. Compared with traditional methods, it has the characteristics of fast processing speed and relatively high diagnostic performance, it has a broad application prospect in cardiovascular imaging (24, 25). Previous AI-ADS studied on the diagnostic accuracy of coronary artery stenoses have mostly focused on patients with conventional cardiac rhythms (26). Han et al. (27) included 50 patients who underwent ICA and CCTA, for the diagnosis of above moderate stenoses, the diagnostic accuracy, sensitivity and specificity were 86%, 88% and 85% for CCTA-AI and 83%, 59% and 94% for CCTA, respectively. Chen et al. (12) reported on 124 patients who underwent ICA and CCTA, the AUC, sensitivity and specificity of DL-model and Read-model based on the segment level were 0.84 and 0.89, 73% and 84%, 96% and 95%, respectively for the diagnosis of above moderate stenoses; Yang et al. (28) studied the diagnostic performance of radiologists with and without AI-ADS for stenoses detection in CAD and found that AUC of radiologists combined with AI-ADS increased from 0.81–0.82 on the patient level and from 0.79–0.81 on the vessel level compared with radiologists alone. Our research was mainly focused on patients with AF. Although CCTA in patients with AF was more challenging, our results of coronary artery diagnosis for AF patients were basically similar to previous research results of using AI in patients with conventional rhythm.

This study had relatively low sensitivity for the diagnosis of coronary stenoses in patients with AF, especially for the diagnosis of above severe stenoses with AI-ADS, due to the false negative result of AI-ADS in the diagnosis process of some vessels near moderate stenoses or near severe stenoses, some studies have also pointed out that AI-ADS has a strong ability to identify above moderate stenoses, but its ability to distinguish between moderate and severe stenoses were not good (27). Previous studies have found that plaque length and calcification score can affect the interpretation of the degree of coronary stenoses (29, 30), Xu et al. (31) pointed out that AI-ADS easily underestimates the coronary stenoses caused by diffused plaques; Xu et al. (32) included 110 patients who underwent CCTA and ICA, the sensitivity and specificity based on segments were 76.8% and 93.7%. The study found that the diagnostic sensitivity positively correlated with calcification burden and diabetes mellitus, and the diagnostic specificity negatively correlated with stenoses severity and calcification burden. In our study, patients with coronary artery stenoses caused by calcified or mixed plaques accounted for 67.03%, and patients with severe stenoses accounted for 64.84%. The sensitivity and specificity of AI-ADS to the diagnosis of above moderate stenoses based on segment level were 63.59% and 95.99%, but the sensitivity to the diagnosis of severe stenoses were 36.92%. However, AI-ADS + radiologist improved the diagnostic performance in patients with atrial fibrillation to a certain extent, and its accuracy, sensitivity and negative predictive value were higher than AI-ADS.

There were some limitations in our study. Firstly, it was a single-center small-sample retrospective study, which needs to be further verified by prospective large-sample clinical trials; Secondly, the included subjects were patients screened in hospital, and the detection rate of stenoses were high, which were prone to selective bias. Thirdly, the CT device in this study only included one 256-slice CT, and the diagnostic performance may not be fully applicable to other types of devices.

## 5 Conclusion

One-beat prospective CCTA combined with SSF1 provides high-quality coronary artery images in patients with AF. AI-ADS can quickly and automatically identify coronary artery images with different degrees of stenoses. However, AI-ADS has a low sensitivity, especially for the diagnosis of severe stenoses. AI-ADS + radiologist further improves the coronary artery diagnosis performance in patients with AF.

## Data Availability

The original contributions presented in the study are included in the article/Supplementary Material, further inquiries can be directed to the corresponding author.
